# Tetrahydrocurcumin Alleviates Metabolic Dysfunction-Associated Steatohepatitis in Mice by Regulating Serum Lipids, Bile Acids, and Gut Microbiota

**DOI:** 10.3390/ijms26030895

**Published:** 2025-01-22

**Authors:** Shang Peng, Moran Meng, Ping Luo, Jiao Liu, Junjun Wang, Yong Chen

**Affiliations:** Hubei Province Key Laboratory of Biotechnology of Chinese Traditional Medicine, National & Local Joint Engineering Research Center of High-Throughput Drug Screening Technology, College of Health Science and Engineering, Hubei University, Wuhan 430062, China; 202411127010188@stu.hubu.edu.cn (S.P.); 20090060@hubu.edu.cn (M.M.); q18171229958@outlook.com (P.L.); liujiao1@hbdxwmh.wecom.work (J.L.)

**Keywords:** tetrahydrocurcumin, lipids, bile acids, gut microbiota, non-alcoholic steatohepatitis

## Abstract

The aim of this study was to investigate the protective effects and potential mechanisms of Tetrahydrocurcumin (THC) on methionine–choline-deficient diet (MCD)-induced MASH in C57BL/6 mice by using multi-omics techniques. The C57BL/6 mice were fed with the MCD for 8 weeks to establish a MASH model, while THC (100 mg·kg^−1^·d^−1^) and obeticholic acid (6.5 mg·kg^−1^·d^−1^) were administered via gavage to the THC group and the positive control group, respectively. The biochemical indexes of the serum and liver were detected using kits. Liver tissue sections were taken to observe the pathomorphological changes. Serum lipid and bile acid contents were measured via LC-MS, and the changes in ileal intestinal flora were detected by 16S rDNA high-throughput sequencing technology. The results revealed that THC significantly attenuated oxidative stress and lipid accumulation in NCTC-1469 cells and relieved hepatic injury and oxidative stress, reduced hepatic TG content, and improved hepatic steatosis in mice. THC alleviated 34 lipid abnormalities caused by the MCD; increased the abundance and diversity of intestinal flora, the ratio of *Firmicutes* to *Bacteroidota*, and the abundance of the probiotic (*Verrucomicrobiota*, *Christensenellaceae*, *Akkermansiaceae*, *Lachnospiraceae*, *Desulfovibrionaceae*); and reduced the abundance of obesity-associated pathogenic flora such as *Firmicutes*. Bile acid analysis showed that THC administration reduced the levels of serum toxic bile acid 7-KDCA and CA. In addition, RT-qPCR studies showed that THC down-regulated the transcript levels of the hepatic lipogenesis-related genes *Srebp1c*, *Acc1*, *Scd1*, and *Fas*, and up-regulated the transcript levels of the hepatic bile acid secretion-related genes *Mrp2* and *Bsep*. The above results suggest that THC may alleviate MCD-induced MASH by downregulating liver *Srebp1c*, *Acc1*, *Scd1*, and *Fas* levels to inhibit lipid synthesis, upregulating *Mrp2* and *Bsep* levels to regulate serum toxic BA levels, up-regulating the abundance of intestinal probiotic flora, and down-regulating the abundance of intestinal harmful bacterial flora. The multi-omics findings from the above study identified potential new mechanisms by which THC alleviates MASH, providing new reference targets for the development of anti-MASH drugs. These results also offer a basis for screening clinical diagnostic biomarkers for MASH and provide new directions for personalized diagnosis and treatment.

## 1. Introduction

Metabolic dysfunction-associated steatotic liver disease (MAFLD) is one of the most common liver diseases worldwide [1], with an estimated prevalence of about 30–35% globally [2]. The progression of metabolic dysfunction-associated steatohepatitis (MASH) is influenced by various factors, including lifestyle choices, circadian rhythm disruption, mitochondrial dysfunction, and gut microbiota dysbiosis. Pathophysiologically, the hallmark of MASH is hepatic steatosis, characterized by the accumulation of triglycerides (TG) in the liver, hepatocyte necrosis, and inflammation [3]. As hepatocyte injury worsens and the inflammatory response intensifies, MASH may progress to liver fibrosis, cirrhosis, and even hepatocellular carcinoma [4]. Currently, the pathogenesis of MASH remains unclear, and the options for therapeutic drugs are still quite limited.

Lipid metabolism disorders are closely associated with MAFLD, obesity, type 2 diabetes, and other metabolic diseases. The abnormal lipid compositions of hepatocytes in MASH patients can lead to lipotoxicity, leading to organelle dysfunction, oxidative stress, chronic inflammation, and apoptosis of liver cells [5]. Disruptions in cholesterol levels or the signal transduction of certain lipids, such as saturated fatty acid (FA), phospholipids, and ceramide, exhibit pro-inflammatory and pro-apoptotic properties, thereby promoting the occurrence and progression of MASH [6,7,8,9].

Bile acids are the end products of cholesterol metabolism and play a crucial role in regulating liver lipid metabolism [10]. Primary bile acids are synthesized in the liver, conjugated with glycine or taurine, secreted into bile, and then released into the intestine to promote lipid absorption, where they are converted into secondary bile acids by the gut microbiota. These bile acids are subsequently reabsorbed into the liver during enterohepatic circulation, inhibiting hepatic bile acid synthesis. The metabolism of bile acids is tightly regulated by negative feedback, and any dysregulation can lead to bile acid accumulation, ultimately causing hepatotoxicity [11]. Studies indicate that dysregulation of bile acids promotes the occurrence of hepatocellular carcinoma driven by MASH in mice [12]. The composition of bile acids in the serum and feces of MAFLD patients differs from that of healthy individuals, with primary bile acid levels continuously increasing with the severity of MAFLD histological changes [13]. Additionally, the gut microbiota plays an important role in regulating MASH by producing short-chain FAs, secondary bile acids, and trimethylamine [14]. Dysbiosis can disrupt energy metabolism homeostasis, leading to hepatic steatosis and changes in intestinal permeability associated with liver inflammation and fibrosis [15]. Specific enzymes produced by the gut microbiota can modify bile acids transported to the intestine and affect their metabolism [16]. The gut microbiota can also activate or inhibit receptors like FXR and TGR5 to regulate bile acid and glucose metabolism as well as overall energy homeostasis [17].

Natural food components have long been recognized for their preventive effects on various chronic diseases; however, many of these compounds lack evidence-based research. Polyphenolic compounds have demonstrated lipid-lowering properties in numerous preclinical studies and some clinical trials [18]. Tetrahydrocurcumin (THC) is the primary metabolite produced during the metabolism of curcumin in vivo. It exhibits various pharmacological activities, including antioxidant, anti-inflammatory, and hypolipidemic effects [19,20]. In oleic acid-induced HepG2 cells, THC can inhibit the expression of lipogenesis related proteins such as sterol regulatory element-binding protein 1 (SREBP-1c), peroxisome proliferator-activated receptor gamma (PPAR-γ), fatty acid synthase (FAS), and fatty acid-binding protein 4 (FABP4). It also upregulated the protein levels of the fatty acid β-oxidation-related factor PPAR-α and reduced oleic acid-induced intracellular lipogenesis in an AMP-activated protein kinase-dependent manner [21]. Furthermore, THC reduced liver lipidosis in C57BL/6 obese mice induced by a high-fat diet, inhibited hepatic steatosis, and significantly decreased the volume of adipocytes. It downregulated the expression of PPAR, CCAAT/enhancer-binding protein α, and downstream fatty acid synthase, while increasing the levels of the negative regulator of lipogenesis, Dlk1/pref-1, thereby regulating lipogenesis in adipose tissue [22]. THC restores lipid phagocytosis by regulating the mTORC1-TFEB pathway in MASH rats and steatotic hepatocytes, thereby reducing lipid accumulation [23].

This study aims to investigate the intervention effects of THC on MASH mice, exploring its impact on serum lipids, bile acids, and gut microbiota diversity in MASH mice. Additionally, through bioinformatics analysis, the study examines the correlations among the above three factors influenced by THC, providing valuable insights into the mechanism by which THC may prevent MASH.

## 2. Results

### 2.1. Effects of THC on Liver Function-Related Biochemical Indicators in MASH Mice

We found that THC reduced TG and ROS levels in NCTC-1469 cells treated with OA and PA, suggesting that THC may have lipid-lowering and oxidative stress-reducing effects (Figure S1). Next, we validated the efficacy of THC in vivo. As shown in Table 1, compared to the MCS group, the mice in the MCD group exhibited significantly higher activity of ALT and AST in serum, as well as increased TG and MDA content in the liver. In contrast, compared to the MCD group, both the OCA- and THC-treatment groups showed significantly reduced activity of ALT and AST in serum, as well as decreased TG and MDA content in the liver. These results suggested that intervention with THC could alleviate liver damage and the accumulation of TG in the livers of MCD-induced MASH mice.

### 2.2. Effects of THC on Liver Histopathology in Mice

**Figure 1:**
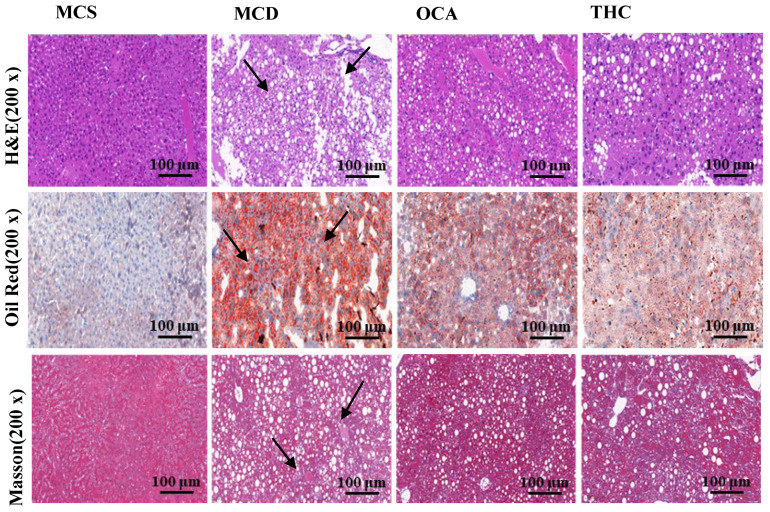
The effects of THC on HE, Oil Red O, and Masson staining of liver tissues in mice (*n* = 7). Black arrows indicate bigger cytoplasmic vacuolation and hepatocellular ballooning, respectively, in HE staining, bigger red lipid droplets in Red Oil O staining, and hepatic fibrosis in Masson staining. For HE staining, the black arrow points to the larger cytoplasmic vacuolation and uneven arrangement of hepatocytes. For Oil Red O staining, the black arrow points to the larger red lipid droplets in the hepatocytes. For Masson staining, the black arrow points to the fibrotic areas in the liver.

As shown in Figure 1, the results of HE staining, Oil Red O staining, and Masson staining revealed that the liver tissue of the MCS group exhibited a normal morphological structure, with tightly arranged hepatocytes and no inflammatory infiltration, fat degeneration, ballooning, or fibrosis. In contrast, the liver tissue of the MCD group showed significant pathological changes, including swollen hepatocytes, ballooning degeneration of necrotic cells, numerous fat vacuoles between hepatocytes, visible fat degeneration, red lipid droplets inside cells, inflammatory cell infiltration, and partial fibrosis of liver tissue. Compared to the MCD group, the THC and OCA groups showed a marked reduction in liver fat degeneration and inflammation, with a significant decrease in the number of lipid droplets. These results indicated that both THC and positive control OCA had the effects of reducing liver lipid accumulation, alleviating hepatic steatosis, and improving lobular inflammation.

### 2.3. Effects of THC on Serum Lipidomics

**Figure 2:**
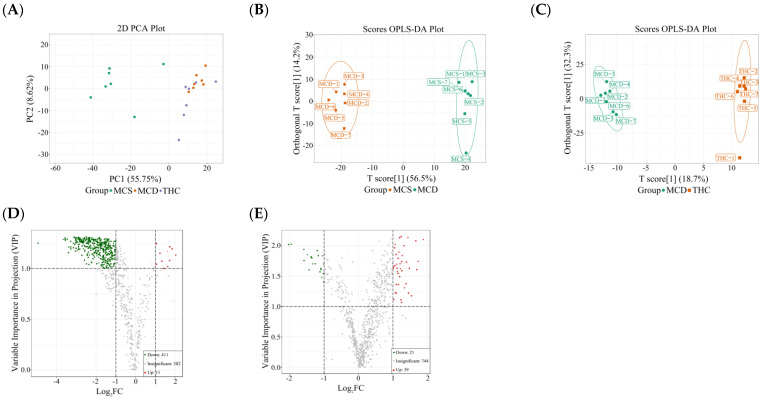

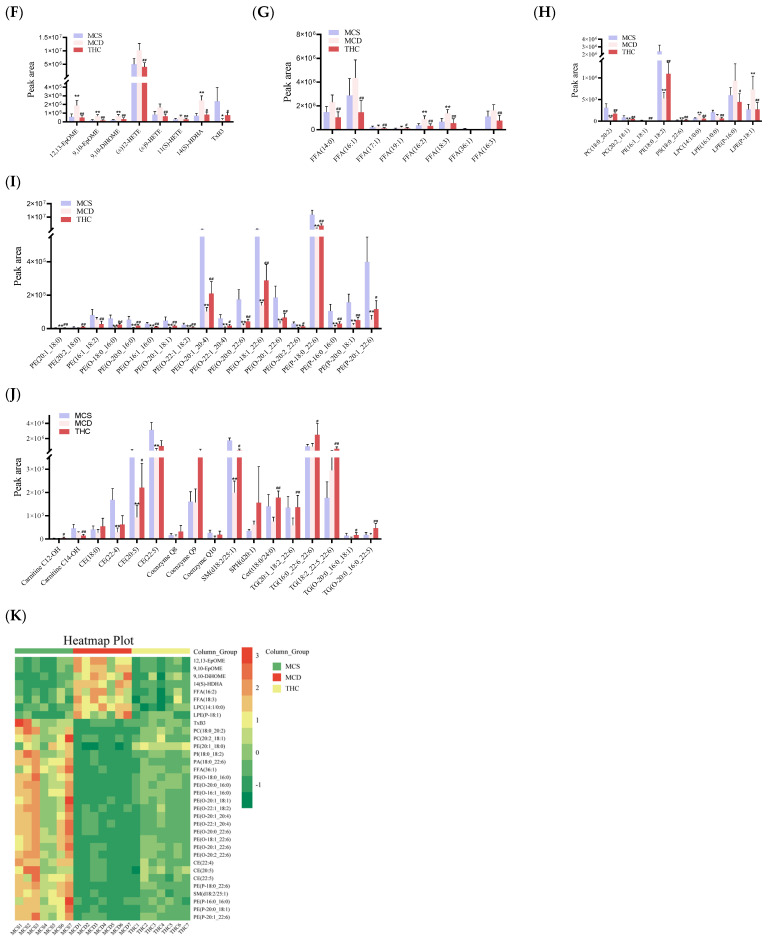
Analysis of the serum lipidomics of the tested mice (*n* = 7). (**A**): A PCA score plot. (**B**): An OPLS-DA score plot between the MCS and MCD groups. (**C**): An OPLS-DA score plot between the MCD and THC groups. (**D**): A volcano plot of lipid metabolites between the MCS and MCD groups. The red dots represent upregulated differential lipids; the green dots represent downregulated differential lipids. (**E**): A Volcano plot of lipid metabolites between the MCD and THC groups. (**F**–**J**): The levels of representative differential lipid species. (**K**): A heat map of differential lipids with significant changes in the MCS, MCD, and THC groups. The redder the color, the higher the relative lipid content; the greener the color, the lower the relative lipid content.* *p* < 0.05, ** *p* < 0.01 versus MCS; ^#^ *p* < 0.05, ^##^ *p* < 0.01 versus MCD group.

A total of 804 lipid compounds were detected in serum, including 105 fatty acyls (FA), 25 sterol lipids (ST), 411 glycerophospholipids (GP), 79 sphingolipids (SP), 3 prenol lipids (PR), and 181 glycerides (GL). The PCA results (Figure 2A) showed a clear distinction between the MCS and MCD groups, indicating significant changes in lipid metabolism in mice induced by MCD. The THC group exhibited a trend of separation from the MCD group, with some overlap, suggesting that THC partially regulated the lipid metabolism alterations induced by the MCD in MASH mice.

To further identify differences in lipid species among the groups, OPLS-DA analysis was performed. The model quality was evaluated using R^2^Y (representing the explanatory power) and Q^2^ (representing the predictive power), with values greater than 0.4 indicating reliable models. As shown in Figure 2B,C, significant differences were observed between the MCS and MCD groups (R^2^Y = 0.994, Q^2^ = 0.962) as well as between the THC and MCD groups (R^2^Y = 0.996, Q^2^ = 0.811).

Differential lipids between groups were further screened based on the VIP values (VIP ≥ 1) and fold change values (FC ≥ 2 or ≤ 0.5). The results, depicted in Figure 2D–J, showed that 422 lipids were significantly altered in the MCD group compared to the MCS group, with 411 down-regulated and 11 up-regulated. Compared to the MCD group, 60 differential lipids were identified in the THC group, of which 21 were down-regulated and 39 were up-regulated lipids.

As shown in Figure 2K, THC treatment alleviated the abnormal changes in 34 lipids among the 422 lipids altered by the MCD. These included four eicosanoids (12,13-EpOME, 9,10-EpOME, 9,10-DiHOME, 14(S)-HDHA), two free FAs (FFA(16:2), FFA(18:3)), one lysophosphatidylcholine (LPC(14:1/0:0)), and one lysophosphatidylethanolamine (LPE(P-18:1)), which were down-regulated. In addition, 2 phosphatidylcholines (PC(18:0_20:2), PC(20:2_18:1)), 16 phosphatidylethanolamines (e.g., PE(20:1_18:0), PE(O-20:0_16:0), PE(O-22:1_20:4)), 1 phosphatidylinositol (PI(18:0_18:2)), 1 phosphatidic acid (PA(18:0_22:6)), 3 cholesteryl esters (CE(22:4), CE(20:5), CE(22:5)), 1 thromboxane B3 (TxB3), 1 free FA (FFA(36:1)), and 1 sphingomyelin (SM(d18:2/25:1)) were up-regulated. These findings suggest that THC has the potential to restore lipid metabolism imbalances induced by the MCD.

**Figure 3:**
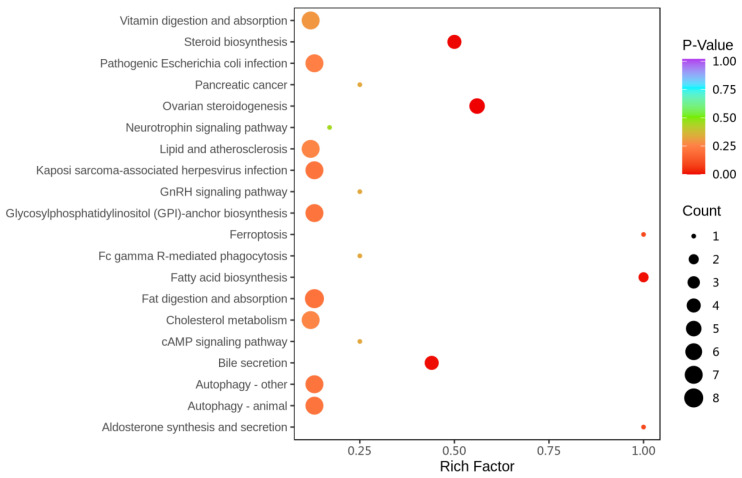
KEGG analysis of differential lipids enriched in pathways (*n* = 7).

The KEGG pathway enrichment analysis identified pathways associated with the 34 differential lipids mentioned above, as shown in Figure 3. These differential lipids were involved in various metabolic pathways. Notably, pathways such as fatty acid biosynthesis, ferroptosis, aldosterone synthesis and secretion, ovarian steroidogenesis, steroid biosynthesis, and bile secretion were highly related to the regulatory effects of THC on lipid metabolism disorders in MASH.

### 2.4. Effects of THC on Composition of Gut Microbiota in MASH Mice

16S rDNA sequencing analysis was conducted on the ileal contents of the mice, yielding a total of 1,394,089 sequencing reads. The alpha diversity index was used to analyze the richness, diversity, and evenness of the microbiota. The Chao1 and ACE indices reflect richness, while the Simpson and Shannon indices represent diversity. As shown in Table 2, compared to the MCS group, the Chao1 and ACE indices in the MCD group were significantly reduced, indicating that the MCD led to a decrease in the richness of the gut microbiota. After THC treatment, the Chao1, ACE, and Shannon indices were significantly increased, suggesting that THC could enhance the richness and diversity of the gut microbiota in MASH mice.

**Figure 4:**
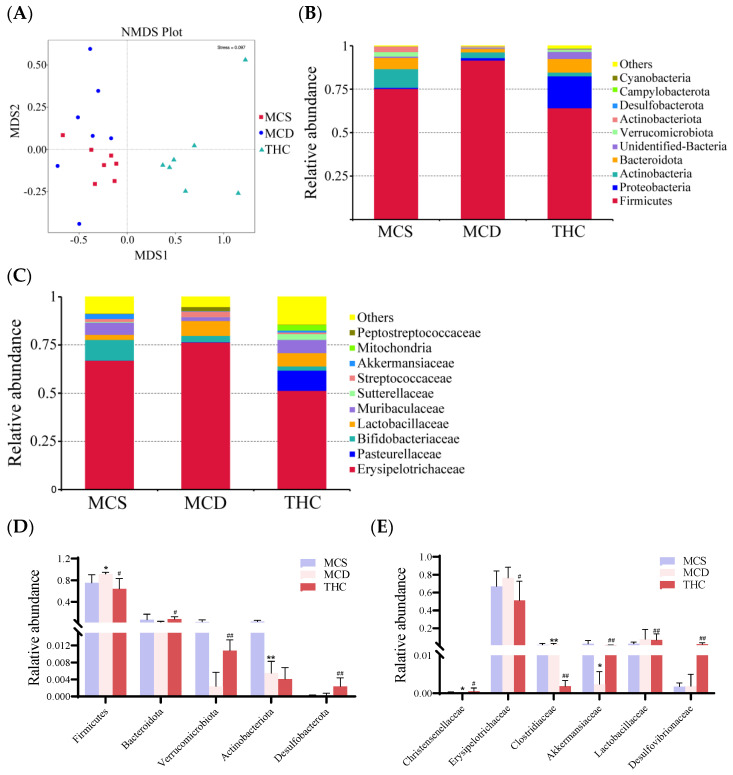
The effects of THC on the composition of gut microbiota in the mice (*n* = 7). (**A**): Non-metric multidimensional scaling (NMDS) on the OTU level. (**B**): Microbiota composition at the phylum level. (**C**): Microbiota composition at the family level. (**D**): Changes in the gut microbiota of mice at the phylum level. (**E**): Changes in the gut microbiota of mice at the family level. * *p* < 0.05, ** *p* < 0.01 versus MCS; ^#^ *p* < 0.05, ^##^ *p* < 0.01 versus MCD group.

Beta diversity analysis was used to compare the composition differences in the gut microbiota in different groups. Non-metric multidimensional scaling (NMDS) analysis showed clear distinctions in the composition of the gut microbiota among the MCS, MCD, and THC groups (Figure 4A).

The top 10 most abundant gut microbiota in the ileal contents of the mice at the phylum and family levels, are shown in Figure 4B,C. At the phylum level, the dominant phyla were *Firmicutes*, *Proteobacteria*, and *Actinobacteria*. Compared to the MCS group, the relative abundance of *Firmicutes* was significantly higher, while *Actinobacteria* was significantly lower in the MCD group (*p* < 0.05). After THC treatment, the relative abundance of *Firmicutes* was significantly reduced (*p* < 0.01), while the relative abundance of *Bacteroidota*, *Verrucomicrobiota*, and *Desulfobacterota* was significantly increased (Figure 4D).

At the family level, *Erysipelotrichaceae*, *Pasteurellaceae*, and *Bifidobacteriaceae* were the predominant families. Compared to the MCS group, the MCD group showed a significant reduction in the abundance of *Christensenellaceae* and *Akkermansiaceae*, with a significant increase in *Clostridiaceae*. In contrast, the THC group had a significant decrease in *Erysipelotrichaceae* and *Clostridiaceae* and a significant increase in *Akkermansiaceae*, *Christensenellaceae*, *Lachnospiraceae*, and *Desulfovibrionaceae* compared to MCD group (Figure 4E).

### 2.5. Effect of THC on Serum Bile Acids in MASH Mice

The levels of 50 bile acids in the serum of mice were measured, and the bile acids with significant differences are shown in Table 3. As the table indicates, the levels of nor-deoxycholic acid (23-DCA), β-muricholic acid (β-MCA), ursodeoxycholic acid (UCA), ω-muricholic acid (ω-MCA), and 7-ketodeoxycholic acid (7-KDCA) were significantly higher in the MCD group than in the MCS group. In contrast, compared to the MCD group, the THC-treated group showed a significant reduction in the levels of 23-DCA, β-MCA, UCA, 7-KDCA, cholic acid (CA), hyodeoxycholic acid (HDCA), and taurodeoxycholic acid (TDCA).

### 2.6. Effect of THC on Genes Related to Fatty Acid Biosynthesis and Bile Acid Homeostasis in Mice

**Figure 5:**
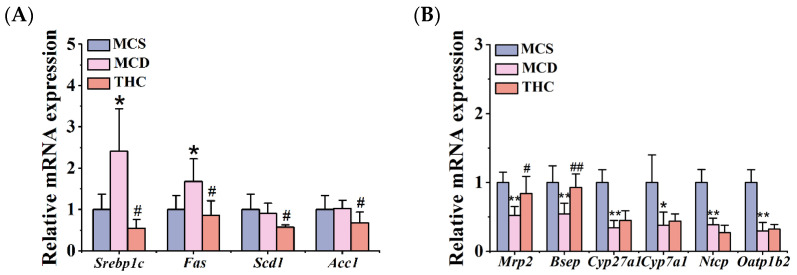
The mRNA expression of lipogenesis- (**A**) and bile acids homeostasis (**B**)-related genes in the MCS, MCD, and THC groups (*n* = 6). * *p* < 0.05, ** *p* < 0.01 versus MCS; ^#^ *p* < 0.05, ^##^ *p* < 0.01 versus MCD group.

To explore the potential therapeutic mechanism of THC on MASH, the mRNA expression levels related to fatty acid biosynthesis and bile acid homeostasis were examined based on the KEGG pathway enrichment results. As shown in Figure 5, the mRNA expression levels of *Srebp1c* and *Fas* in the MCD group were significantly higher than those in the MCS group, while the mRNA expression levels of *Cyp27a1*, *Cyp7a1*, *Bsep*, *Mrp2*, *Ntcp*, and *Oatp1b2* were significantly lower in the MCD group. THC treatment notably reversed the levels of Srebp1c and down-regulated its downstream genes *Acc1*, *Scd1*, and *Fas*, and up-regulated the mRNA expression of *Mrp2* and *Bsep*.

### 2.7. Correlation Analysis of Differentially Changed Lipids, Bile Acids, and Microbiota

**Figure 6:**
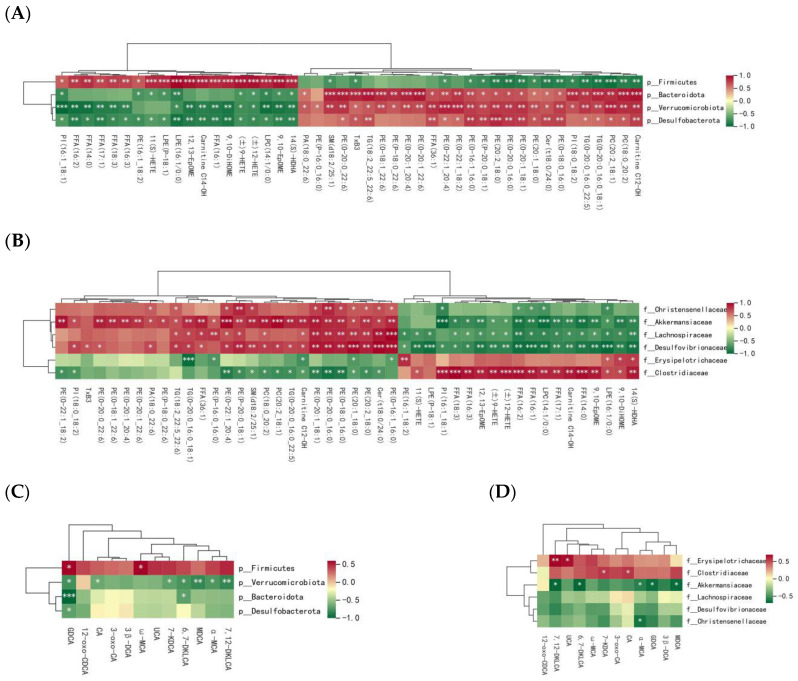
The correlations between GM and serum lipids or BAs at the phylum level (**A**,**C**) and family level (**B**,**D**) in THC-treated MCD mice. * indicates *p*  <  0.05, ** indicates *p*  <  0.01, and *** indicates *p*  <  0.001.

The results of the correlation analysis are shown in Figure 6. At the phylum level, the *Firmicutes* phylum was negatively correlated with Cer(t18:0/24:0), SM(d18:2/25:1), 10 types of PE, and Carnitine C12, while it was positively correlated with LPC(14:1/0:0), PI(16:1_18:1), LPE(16:1/0:0), 9,10-DiHOME, Carnitine C14-OH, (±)9-HETE, (±)12-HETE, 9,10-EpOME, 12,13-EpOME, and 14(S)-HDHA. The *Bacteroidota* phylum was negatively correlated with PI(16:1_18:1), PE(16:1_18:2), 11(S)-HETE, LPE(P-18:1), LPE(16:1/0:0), (±)9-HETE, (±)12-HETE, LPC(14:1/0:0), 9,10-EpOME, and 14(S)-HDHA, and positively correlated with SM(d18:2/25:1), TxB3, TG(18:2_22:5_22:6), 13 types of PE, FFA(36:1), Cer(t18:0/24:0), PI(18:0_18:2), PC(18:0_20:2), PC(20:2_18:1), TG(O-20:0_16:0_22:5), TG(O-20:0_16:0_18:1), and Carnitine C12-OH. The *Verrucomicrobiota* phylum was negatively correlated with PI (16:1_18:1), FFA (16:2), and FFA(14:0), and positively correlated with PA(18:0_22:6), PE(P-16:0_16:0), SM(d18:2/25:1), and 27 other lipids. The *Desulfobacterota* phylum was negatively correlated with PI (16:1_18:1), FFA (16:2), and FFA(14:0), and positively correlated with PE(O-20:0_22:6), TxB3, and TG(18:2_22:5_22:6) (Figure 6A). As shown in Figure 6C, *Firmicutes* was positively correlated with GDCA and ω-MCA, while *Verrucomicrobiota* was negatively correlated with GDCA, 7-KDCA, 6, 7-DKLCA, MDCA, α-MCA, 7, 12-DKLCA, and CA. *Bacteroidota* was negatively correlated with GDCA and 6, 7-DKLCA, and *Desulfobacterota* was negatively correlated with GDCA.

At the family level, *Christensenellaceae* was positively correlated with PA(18:0_22:6), TG(18:2_22:5_22:6), 8 types of PE, Cer(t18:0/24:0), and SM(d18:2/25:1), and negatively correlated with PI(16:1_18:1), FFA(16:2), FFA(16:1), LPC(14:1/0:0), and LPE(16:1/0:0). *Akkermansiaceae* was positively correlated with 26 lipids, including PE (O-22:1_18:2) and PI (18:0_18:2), and negatively correlated with 16 lipids, including PI (16:1_18:1), FFA (18:3), and FFA(16:3). *Lachnospiraceae* was positively correlated with 15 lipids, including TG (18:2_22:5_22:6) and FFA (36:1), and negatively correlated with 18 lipids, including PE (16:1_18:2) and 11(S)-HETE. *Desulfovibrionaceae* was positively correlated with 21 lipids, including PI (18:0_18:2) and TxB3, and negatively correlated with 19 lipids. *Erysipelotrichaceae* was positively correlated with lipids like TG (O-20:0_16:0_18:1) and negatively correlated with PE (16:1_18:2) and 10-DiHOME. *Clostridiaceae* was positively correlated with 15 lipids, including PE (O-18:0_16:0), and negatively correlated with 17 lipids (Figure 6B).

Finally, *Erysipelotrichaceae* was positively correlated with 7,12-DKLCA and UCA, and *Clostridiaceae* with 7-KDCA and CA; *Akkermansiaceae* was negatively correlated with 7,12-DKLCA, 6,7-DKLCA, α-MCA, GDCA, and MDCA; and *Christensenellaceae* was negatively correlated with α-MCA (Figure 6D).

## 3. Discussion

Choline is a crucial nutrient in lipid metabolism, playing a role in processes such as fatty acid metabolism, cholesterol metabolism, and lipid transport through methylation reactions in the liver. Choline can also influence the composition and metabolism of the gut microbiota. Choline deficiency may alter the structure of gut microbial communities and the production of their metabolites, impacting liver inflammation and fat metabolism, thereby exacerbating the progression of MASH. The MCD model is widely used for drug screening for MASH, as it closely resembles the pathological liver changes seen in MASH patients, with advantages such as a short modeling period and high success rate, making it a classic MASH animal model.

Although studies have shown that THC has potential therapeutic effects on liver steatosis in mice with obesity and MAFLD [24,25], little is known about its effects on the changes in lipid metabolism, the gut microbiota, and bile acids in MASH. In this study, we found that THC administration significantly reduced serum ALT and AST activity and hepatic TG and MDA levels, ameliorated hepatic histological lesions, regulated the levels of lipids and BA, and changed the composition and abundance of the gut microbiota in MCD-induced MASH mice, which, in turn, improves MASH. To date, no clinical studies on THC for the treatment of MASH have been reported. However, as a major metabolite of curcumin in vivo, it exhibits similar pharmacological activities to curcumin and has better bioavailability and stability than curcumin [26]. Clinical studies have shown that supplementation with curcumin can significantly reduce levels of ALT, AST, FBI, TC, and TG in patients with MAFLD [27]. Curcumin also significantly reduced the ratio of *Firmicutes* to *Bacteroidetes* in NASFL patients and significantly increased the abundance of Bacteroidetes, and effectively alleviated MASH [28]. Moreover, curcumin can improve type 2 diabetes and other metabolic syndromes, significantly reducing serum levels of TG, HDL-C, and MDA in individuals with type 2 diabetes [29,30]. The results of this study found that THC reduced serum levels of ALT and AST, and liver MDA and TG in MASH mice, and also decreased the abundance of *Firmicutes* and increased the abundance of *Bacteroidetes* in the ileal contents. These effects are similar to those of curcumin in clinical studies, suggesting that THC may be a promising candidate compound for the treatment of MASH.

Dyslipidemia is a hallmark of various metabolic diseases, including obesity, hyperlipidemia, and fatty liver, and is closely related to the occurrence and progression of MASH [5]. Therefore, serum lipidomics analysis can provide valuable insights for MASH research. Free fatty acids (FFAs) not only promote the excessive accumulation of diacylglycerols (DGs) and TGs in the liver, but also cause insulin resistance and induce obesity [31]. In addition, studies have shown that unsaturated FAs play an important role in the development of steatosis and inflammation [32]. Eicosanoids, oxidative products of polyunsaturated FA, are potent mediators of inflammation [33]. For example, 9, 10-DiHOME can induce oxidative stress, inflammation, and adipogenesis in in vitro models [34,35,36], and 12-HETE, a metabolite of arachidonic acid, has widespread and significant effects on cell signaling, metabolism, and inflammatory responses [37]. We found that most eicosanoids and FFAs increased significantly in the MCD group, while THC intervention significantly reduced the levels of nine eicosanoids and seven FFAs. TXB3, an eicosanoic acid produced from the n-3 series of eicosapentaenoic acids, is a bioactive lipid mediator with anti-inflammatory effects. PE is a precursor for synthesizing PC, and reduced PE levels lead to impaired PC synthesis, resulting in decreased very-low-density lipoprotein synthesis and abnormal lipid metabolism [38]. Our study demonstrated that the levels of PE and PC significantly decreased in MCD-induced MASH mice, while THC intervention significantly reduced the elevated levels of TXB3 and 17 PEs induced by the MCD. These findings suggested that the alleviating effect of THC on MCD-induced MASH is closely related to its regulatory effects on serum FFA, eicosanoid, and PE levels.

In recent years, an increasing number of studies have reported the impact of the gut microbiota on MASH. The gut microbiota influences MASH progression by promoting host energy absorption, altering bile acid and choline metabolism, thereby promoting inflammation and insulin resistance [15,39,40]. Our study showed that the MCD reduced the diversity of the gut microbiota, while THC enriched microbiota diversity and alleviated the dysbiosis of the mice in MCD group. It has been reported that an increase in the abundance of *Firmicutes* and in the ratio of *Firmicutes* to *Bacteroidetes* is a typical feature of obese mice, and obesity is a risk factor for metabolic syndrome [41]. Our research found that THC significantly reduced the relative abundance of *Firmicutes* and increased the relative abundance of *Bacteroidetes* at the phylum level, thereby lowering the *Firmicutes*-to-*Bacteroidetes* ratio, indicating that THC played an important role in regulating obesity-related microbial imbalances. Additionally, THC increased the relative abundance of *Verrucomicrobia*, which is positively associated with health and can alleviate obesity and insulin resistance [42]. *Verrucomicrobia* also helps prevent fatty liver by regulating the expression of genes related to liver fat synthesis and inflammation, maintaining intestinal homeostasis [43]. *Akkermansiaceae* is a potential probiotic that alleviates endotoxemia, inflammation, and immune disorders, and improves gut barrier function [44], and its abundance is negatively correlated with overall fat content [45]. The relative abundance of *Christensenellaceae* is inversely correlated with body mass index (BMI) [46] and negatively associated with obesity and inflammatory bowel disease [47]. Our study found that the MCD decreased the levels of *Christensenellaceae* and *Akkermansiaceae*, while THC significantly alleviated this decline. *Desulfovibrionaceae*, an anti-inflammatory and sulfate-reducing bacterium that produces H_2_S, has anti-inflammatory and gastrointestinal mucosal repair effects [42,48]. Butyrate, which improves intestinal barrier function, reduces inflammation, and lowers endotoxin levels, playing a protective role in MCD-induced MASH, and *Desulfovibrioideae* is an important source of butyrate [49]. Our study also found that THC increased the relative abundance of *Lachnospiraceae* and *Desulfovibrioideae*. Therefore, reducing harmful microbiota and increasing beneficial microbiota may be a potential factor in THC’s ability to ameliorate MASH.

Bile acids play an essential role in lipid absorption, and they also act as ligands for various receptors to regulate glucose and lipid homeostasis. Bile acids and the pathways they participate in are important targets for MASH treatment. The levels of serum bile acid and secondary bile acids with hydrophobicity and cytotoxicity are higher in patients with MASH, which may be linked to liver injury and MASH pathogenesis [50]. CA is a hydrophobic bile acid that can disrupt mitochondrial electron transport chains, leading to oxidative stress [51]. High concentrations of circulating CA are also associated with liver inflammation and hepatocyte ballooning [52]. An increase in CA level has also been associated with increased Firmicutes and decreased Bacteroidetes levels, posing a risk for obesity [53]. High levels of 7-KDCA in the gut promote the hepatic synthesis and fecal excretion of bile acid in patients with bile acid-related diseases by inhibiting the FXR/FGF19 signaling pathway [54]. We just found that THC reduced the levels of toxic bile acids such as CA and 7-KDCA in serum, suggesting that THC may reduce the accumulation of toxic bile acids in the blood.

To further explore the mechanism by which THC affects lipid metabolism and bile acid homeostasis, we examined the expression of genes related to fatty acid biosynthesis and bile acid homeostasis based on the KEGG pathway enrichment results. SREBP1c is a sterol regulatory element-binding protein that promotes lipid deposition and lipid metabolic disorders, involved in the transcription of FAS and ACC, thereby regulating the levels of TC and TG in the liver. Our results showed that THC significantly down-regulated the mRNA expression of the lipogenic genes *Srebp1*, *Acc1*, *Scd1*, and *Fas*, inhibiting lipid synthesis. Bile acid biosynthesis involves both the classical and alternative pathways. Under normal conditions, more than 75% of bile acids are produced via the classical pathway regulated by enzymes such as CYP7A1 and CYP8B1, while the alternative pathway is catalyzed by CYP27A1. Bile acids are conjugated with taurine or glycine and transported by transport proteins such as *BSEP* and *MRP2*, then form micelles with substances such as cholesterol and phospholipids, and are stored in the gallbladder in the form of bile. After food intake, bile acids are secreted into the duodenum, stimulated by cholecystokinin, to perform the physiological functions of the emulsification and absorption of lipids. About 95% of BAs are reabsorbed at the end of the ileum, secreted into the portal vein via the basolateral bile acid transporter MRP2, etc., and then circulated to the liver, where they are taken up into hepatocytes by NTCP and OATP1, with only a small portion of the unabsorbable bile acids being excreted from the body. These bile acids are in dynamic balance with the biosynthesis of bile acids, leading to the homeostasis of bile acids in organisms. The RT-qPCR results in our study showed that THC significantly up-regulated the mRNA levels of the bile acid efflux transporters *Mrp2* and *Bsep*, promoting bile acid excretion from the liver to the gallbladder. These findings suggested that THC may regulate lipid synthesis and bile acid homeostasis by down-regulating lipogenesis-related genes and up-regulating bile acid efflux-related genes, thus having protective effects in MASH mice.

It is well known that there is a close relationship between the gut microbiota and lipid and bile acid metabolism. Gut microbes can regulate the biotransformation of bile acids via bile salt hydrolases and 7α/β dehydroxylases [55], as well as modifying bile acids via reactions such as esterification, desulfurization, and differential isomerization, increasing the diversity of the bile acid pool within the host [56]. The gut microbiota can also regulate bile acid synthesis and lipid metabolism by affecting the hepatic FXR signaling pathways [49]. On the other hand, bile acids can inhibit the growth of bile acid-sensitive bacteria, thereby modulating the structure of the gut microbiota [57]. Many studies have confirmed that the gut microbiota can regulate lipid and bile acid levels in vivo. Velagapudi found that the gut microbiota altered the types of PC and TG in the serum of mice and regulated lipid metabolism in the serum, adipose tissue, and liver [58]. *Akkermansia muciniphila* was found to produce over 200 lipids, including TG, PE, PC, and SM [59]. And *Akkermansia muciniphila* can effectively prevent fatty liver disease caused by HFD by regulating the liver fat synthesis gene SREBP [42]. *Bacteroides ovatus* treatment down-regulated the levels of genes involved in de novo lipogenesis, such as *Srebfl*, *Acaca*, *Scd1*, and *Fasn* [60]. The correlation analysis in this study suggested that the regulatory trend of THC on beneficial gut microbiota was consistent with that on beneficial serum lipids and bile acids, indicating that THC may regulate the lipid and bile acid contents in mice by modulating the gut microbiota, thereby alleviating MASH. However, the specific inter-regulatory mechanisms need to be further studied.

This study employed a multi-omics approach to identify key disease-related differential lipids and bile acids, such as 9, 10-DiHOME and 7-KDCA, which can provide references for screening clinical diagnostic biomarkers for MASH. This is crucial for disease early warning, diagnosis, and prognostic monitoring. Additionally, through changes in differential microbiota, such as an increase in beneficial bacteria like *Bacteroidota*, *Verrucomicrobiota*, and *Desulfobacterota*, and a decrease in harmful bacteria like Firmicutes, a deeper understanding of the composition of the individual microbiome and its impact on health can be gained, offering new directions for personalized treatment. At the same time, based on the effects of THC on lipids, bile acids, and the gut microbiome, this study provides a research foundation for discovering new mechanisms by which THC improves MASH, offering new reference targets for the development of anti-MASH drugs.

However, our research still has some limitations that need to be addressed in future research. Although the MCD-induced MASH model is an effective model for inducing MASH, especially for preliminary drug screening or investigating certain fundamental mechanisms of MASH, and can present liver pathology, the mice did not exhibit obesity or hyperglycemia, and their body weight and blood glucose levels were significantly lower compared to the control mice. Therefore, this model does not fully replicate the human MASH condition [61]. Due to the model’s limitations, it is still necessary to complement it with other models (such as high-fat diet models, diabetes-combined models, etc.) for more comprehensive research. This will allow for a better simulation of the pathological features and pathogenesis of human MASH. In addition, this study only used male mice. Although we chose male C57 mice because they were more sensitive to diet-induced MASH conditions [62,63], and our focus was on determining whether THC would alleviate MASH, it is still worth exploring the additional benefits of THC in female mice. Future research should investigate the effects of THC on female mice to determine its impact and potential mechanisms, as well as explore other potential sex differences in the response to THC. In addition, while this study examined the effects of THC on lipids, bile acids, and the gut microbiome, along with their correlations, the pathological impacts of these changes in lipids, bile acids, and the microbiota on MASH still require further validation. The underlying molecular mechanisms need to be explored in greater depth.

In conclusion, this study demonstrated that THC can alleviate MASH, and these beneficial effects in MASH mice were mainly achieved by regulating lipid metabolism, gut microbiota diversity and composition, and bile acid homeostasis. Given that this study focused on the changes in the composition of serum lipids, serum bile acids, and the gut microbiota in MCD mice, as well as their correlations, the molecular mechanisms of specific changes have not been thoroughly explored. However, there is a high correlation between changes in the gut microbiota, lipids, and bile acids and metabolic diseases, especially MASH. Therefore, this study still provides valuable insights for further investigation into the mechanisms of the anti-MASH effect of THC. In the future, we will conduct in-depth exploration of the mechanisms by which THC improves MASH, such as using THC-regulated differential lipids, bile acids, and the gut microbiota to treat MCD mice to investigate whether it can improve MASH. At the same time, based on the concept of personalized microbiome therapy, future research could also develop specific probiotics for MASH, beneficial lipid metabolites, or inhibitors targeting specific pathogenic microorganisms and metabolites.

## 4. Methods and Materials

### 4.1. Materials

Obercholic (CAS: 459789-99-2) acid and THC (CAS: 36062-04-1, purity ≥ 98%) were purchased from Shanghai Yuanye Biotechnology Co., Ltd. (Shanghai, China). Aspartate aminotransferase (AST), alanine aminotransferase (ALT), TG, and malondialdehyde (MDA) assay kits were purchased from Nanjing Jiancheng Bioengineering Institute (Nanjing, China). Bile acid standards were obtained from CNW (Osnabrück, Germany). Lipid standards (12:0 Lyso PC(CAS: 20559-18-6), Cer(d18:1/4:0) (CAS: 74713-58-9), PC(13:0/13:0) (CAS: 71242-28-9), DG(12:0/12:0) (CAS: 60562-15-4), TG(17:0/17:0/17:0) (CAS: 2438-40-6)) were sourced from Shanghai Zhenzhun Biotechnology Co., Ltd. (Shanghai, China). Phusion high-fidelity PCR master mix (with GC Buffer) was purchased from New England Biolabs (Ipswich, MA, USA). Ribonuclease A was obtained from Promega (San Diego, CA, USA). Library preparation kits (TruSeq^®^ DNA PCR-Free Sample Preparation Kit) were sourced from Illumina (Madison, WI, USA); SYBR Green I PCR kits were purchased from Bio-Rad (Hercules, CA, USA); and the ReverTra Ace qPCR RT Master Mix with gDNA Remover kit was obtained from Toyobo (Osaka, Japan). Gel extraction kits were purchased from Qiagen (Düsseldorf, Germany).

### 4.2. Animals, Diets, and Treatments

Twenty-eight male C57BL/6 mice (SPF grade, 19–23 g) were purchased from the Hubei Provincial Center for Disease Control and Prevention (SCXK (E) 2017-0012). All mice were housed in the SPF animal facility of Hubei University at a temperature of 20–25 °C and humidity of 55–65%, with a 12 h light–dark cycle. All animal experimental protocols were approved by the Ethics Committee of Hubei University (Approval No: 20220038). After one week of acclimatization, mice were randomly divided into the methionine–choline-sufficient (MCS) group, MCD group, positive control group, and THC-treatment group, with seven mice in each group. The MCS group was fed with the MCS diet, while the model, positive control, and THC-treatment groups were fed with the MCD, with all mice given free access to water and food. The THC-treatment and positive control groups were administered THC (100 mg·kg^−1^·d^−1^) and OCA (6.5 mg·kg^−1^·d^−1^) via gavage daily, while normal and MCD group mice received an equal volume of 0.5% CMC-Na solution. After 8 weeks, mice were fasted for 12 h, blood was collected from the orbital venous plexus under anesthesia, and then the mice were euthanized via cervical dislocation. Liver tissues were rapidly collected and temporarily frozen in dry ice, and then transferred toan environment of −80 °C.

### 4.3. Serum and Liver Biochemical Indicators

A portion of serum was taken to measure ALT and AST activities according to the instructions of the kits. Some liver tissue was weighed and mixed with nine times its volume of pre-cooled saline to prepare a 10% liver homogenate, which was then used to determine TG and MDA levels using the test kits.

### 4.4. Liver Histopathology

The left lobe of liver tissue from each mouse was selected, fixed rapidly in 4% paraformaldehyde for 24 h, and subjected to HE and Masson staining. And the liver tissues were frozen and sectioned into 10 μm slices for Oil Red O staining, with the degree of hepatic steatosis and fibrosis observed under an optical microscope.

### 4.5. Serum Lipidomics Analysis

Fifty microliters of serum were mixed with 1 mL of extraction solution containing methyl tert-butyl ether, methanol, and internal standard. The mixture was vortexed for 15 min; then, 200 μL of deionized water was added and vortexed for 1 min. After centrifugation at 12,000 r·min^−1^ for 10 min at 4 °C, 200 μL of the supernatant was taken and dried. Then, the residue was dissolved by the initial mobile phase for LC-MS/MS analysis. Equal volumes from each sample were combined to create quality control samples, which were inserted every 10 samples during the analysis to monitor reproducibility.

UPLC (ExionLC™ AD) coupled with MS/MS (QTRAP^®^ 6500+) was used for the analysis of serum lipids. The column used was a Thermo Accucore™ C30 column (100 mm × 2.1 mm, 2.6 μm). Mobile phases A and B were acetonitrile–water (60:40) and acetonitrile–isopropanol (10:90, containing 0.1% formic acid and 10 mmol/L ammonium formate), respectively. The gradient elution program was as follows: 0 min, 20% B; 0–2 min; 30% B; 2–4 min: 60% B; 4–9 min: 85% B; 9–14 min: 90% B; 14–15.5 min, 95% B; 15.5–17.5 min, 20% B; 17.5–20 min, 20% B. The flow rate was 0.35 mL/min, the column temperature was 45 °C, and the injection volume was 2 μL. MS/MS detection was performed in MRM modes. The ESI source temperature was 500 °C, with ion spray voltages of 5500 V (positive ion) and −4500 V (negative ion). The ion source parameters were set to gas1 45 psi, gas2 55 psi, curtain gas 35 psi, and the collision-induced dissociation (CID) parameter was set to Medium.

Raw MS data were collected using Analyst 1.6.3 software and imported into the MWDB database. Qualitative analysis was performed based on retention time, ion pairs, and secondary MS data. The characteristic ions of each substance were screened using triple-quadrupole mass spectrometry, and total ion chromatograms of these characteristic ions were integrated and peak-area-corrected. The peak area of each peak represented the relative content of the substance.

R software (www.r-project.org) was used to analyze the mass spectrometry data, including principal component analysis (PCA), orthogonal partial least squares-discriminant analysis (OPLS-DA), clustering analysis, and metabolic pathway analysis.

### 4.6. Serum Bile Acid Analysis

A total of 50 μL of serum was mixed with 10 μL of internal standard working solution at a concentration of 1 mmol·mL^−1^ with 200 μL of methanol. The mixture was vortexed at 2500 rpm for 10 min and placed in a −20 °C freezer for 10 min. After that, the sample was centrifuged at 12,000 rpm for 10 min at 4 °C. The supernatant was dried, and the residue was reconstituted in 100 μL of 50% methanol–water solution.

Bile acids in serum were detected using UPLC (ExionLC™ AD) coupled with MS/MS (QTRAP^®^ 6500+). The chromatographic column used was a Waters ACQUITY UPLC HSS T3 C18 column (100 mm × 2.1 mm, 1.8 µm). The mobile phase consisted of phase A (ultrapure water containing 0.01% acetic acid and 5 mmol ammonium acetate) and phase B (acetonitrile containing 0.01% acetic acid). The gradient elution program was as follows: 0 min: 5% B; 0–0.5 min: 40% B; 0.5–4.5 min: 50% B; 4.5–7.5 min: 75% B; 7.5–10 min: 95% B; 10–12 min: 5% B. The flow rate was 0.35 mL·min^−1^, the column temperature was 40 °C, and the injection volume was 3 μL. Detection was performed in MRM mode for both positive and negative ions. The ESI source temperature was 550 °C, with an ion spray voltage of −4500 V and curtain gas pressure of 35 psi.

Raw data were collected using Analyst 1.6.3 software and imported into the MWDB database. Qualitative analysis was performed based on retention time, ion pair information, and secondary MS data. For the quantitative analysis, characteristic ions for each bile acid were selected using triple-quadrupole mass spectrometry, and total ion chromatograms were integrated and peak-area-corrected. The content of each bile acid was calculated using the linear equation from the standard curve.

### 4.7. Analysis of 16S Gene-Targeted Sequencing of Intestinal Microbiota

The total genomic DNA from the ileal contents of mice was extracted using the CTAB method. After determining the purity and concentration of the extracted DNA, the samples were diluted with sterile water to a concentration of 1 ng·μL^−1^. The 16S V3–V4 region was selected for amplification using the following primer sequences: forward primer 341F (CCTAYGGGRBGCASCAG), reverse primer 805R (GGACTACNNGGGTATCTAAT). PCR amplification was performed in a 15 µL reaction mixture containing 0.2 µM of both forward and reverse primers, and 10 ng of template DNA. The PCR program was as follows: Initial denaturation at 98 °C for 1 min; 30 cycles at 98 °C for 10 s, 50 °C for 30 s, and 72 °C for 30 s; final extension at 72 °C for 5 min. The PCR products were examined via 2% agarose gel electrophoresis, and the target bands were recovered. Library construction was performed using the TruSeq^®^ DNA PCR-Free Sample Preparation Kit. The constructed libraries were quantified using Qubit and Q-PCR. After confirming the library quality, sequencing was performed on the NovaSeq6000 platform (Illumina, San Diego, CA, USA).

Sequences with a similarity of 97% were clustered into OTUs (Operational Taxonomic Units), and the representative sequences were selected based on the most frequent sequence. These representative sequences were annotated using the SILVA database, identifying the microbial communities in the samples at the taxonomic level. The sample data were normalized, and α-diversity and β-diversity analyses were performed using Qiime software (V1.9.1, http://qiime.org/scripts/split_libraries_fastq.html (accessed on 15 January 2025)).

### 4.8. Real-Time q-PCR

Approximately 50 mg of liver tissue was collected from each mouse for total RNA extraction. The extracted RNA was resuspended in 100 μL of RNase-free DEPC-treated water. The concentration and quality of RNA were assessed using agarose gel electrophoresis and a micro-nucleic acid detector. Double-stranded cDNA was synthesized using a reverse transcription kit.

Real-time q-PCR was performed using the SYBR Green dye method, with β-actin serving as the internal reference gene. The relative expression of target genes was calculated using the 2^−ΔΔCt^ method. The primer sequences are provided in Table 4.

### 4.9. Data Analysis

All results are expressed as the mean ± standard deviation. The statistical analysis of the experimental data was performed using SPSS 25.0. The comparison of means between two groups was conducted using an independent samples *t*-test. To prevent the overfitting of the OPLS-DA model, response ordering tests (200 random permutations) and cross-validation were used to evaluate the model’s quality. Spearman correlation analysis was used to evaluate the relationship between the gut microbiota, serum lipids, and bile acids. A value of *p* less than 0.05 was considered statistically significant.

## Figures and Tables

**Table 1:** Effects of THC on biochemical indicators related to liver function in mice (*n* = 7).

	ALT (U/L)	AST (U/L)	MDA (nmol/mg)	TG (nmol/mg)
MCS	11.83 ± 2.25	24.78 ± 5.67	0.73 ± 0.29	0.19 ± 0.10
MCD (vs. MCS)	149.46 ± 60.56 (*p* = 0.0026)	53.05 ± 13.44 (*p* = 0.0008)	6.58 ± 2.40 (*p* = 0.0018)	0.85 ± 0.23 (*p* = 0.00014)
THC (vs. MCD)	74.26 ± 13.90 (*p* = 0.0278)	35.83 ± 15.28 (*p* = 0.0452)	2.96 ± 1.56 (*p* = 0.0007)	0.50 ± 0.26 (*p* = 0.021)
OCA (vs. MCD)	104.45 ± 28.29 (*p* = 0.0993)	37.65 ± 11.74 (*p* = 0.0452)	3.64 ± 1.91 (*p* = 0.041)	0.57 ± 0.20 (*p* = 0.044)

**Table 2:** The effects of THC on the α diversity of mice (*n* = 7).

Group	Chao1	ACE	Shannon	Simpson
MCS	213.73 ± 34.51	216.44 ± 34.48	2.88 ± 0.58	0.73 ± 0.12
MCD	168.32 ± 17.25 *	173.48 ± 15.91 *	2.56 ± 0.28	0.70 ± 0.09
THC	460.26 ± 100.89 ^#^	466.59 ± 104.83 ^#^	3.58 ± 0.70 ^#^	0.74 ± 0.10

* *p* < 0.05 versus MCS, ^#^ *p* < 0.05 versus MCD group.

**Table 3:** Effect of THC on serum bile acids of tested mice (*n* = 7).

Class	MCS/ng·mL^−1^	MCD/ng·mL^−1^	THC/ng·mL^−1^
23-DCA	24.85 ± 18.84	308.11 ± 125.62 **	164.36 ± 100.07 ^#^
β-MCA	821.92 ± 815.48	2433.77 ± 1484.48 *	1172.55 ± 480.97
ω-MCA	1747.67 ± 1192.60	4581.38 ± 2654.89 *	1952.00 ± 1331.20
7-KDCA	894.78 ± 818.95	3167.95 ± 1981.42 *	1981.42 ± 454.07 ^#^
UCA	15.79 ± 9.17	96.60 ± 61.64 *	28.16 ± 17.01 ^#^
CA	1447.63 ± 1373.38	2086.49 ± 982.95	1011.29 ± 447.23 ^#^
HDCA	55.26 ± 47.95	50.10 ± 11.03	26.55 ± 15.15 ^##^
TDCA	401.30 ± 374.15	285.09 ± 80.5	188.67 ± 42.26 ^#^

* *p* < 0.05, ** *p* < 0.01 versus MCS; ^#^ *p* < 0.05, ^##^ *p* < 0.01 versus MCD group.

**Table 4:** RT-qPCR primer sequences.

Gene Name	Forward Primer	Reverse Primer
*β-actin*	AACCGTGAAAAGATGACCCAGAT	CACAGCCTGGATGGCTACGTA
*Cyp7a1*	GGGAATGCCATTTACTTGGA	GTCCGGATATTCAAGGATGC
*Cyp27a1*	CTATGTGCTGCACTTGCCC	GGGCACTAGCCAGATTCACA
*Mrp2*	TCCAGGACCAAGAGATTTGC	TCTGTGAGTGCAAGAGACAGGT
*Bsep*	CCAGAACATGACAAACGGAA	AAGGACAGCCACACCAACTC
*Ntcp*	AGGGGGACATGAACCTCAG	TCCGTCGTAGATTCCTTTGC
*Oatp1b2*	ACCAAACTCAGCATCCAAGC	TAGCTGAATGAGAGGGCTGC
*S* *rebp1c*	CACTTCTGGAGACATCGCAAAC	TGGTAGACAACAGCCGCATC
*Fas*	GGCACCTATGGCGAGGACTT	GCCCTCCCGTACACTCACTC
*Scd1*	TACTACAAGCCCGGCCTCC	CAGCAGTACCAGGGCACCA
*Acc1*	GGCAGCAGTTACACCACATAC	TCATTACCTCAATCTCAGCATAGC

## Data Availability

Data will be made available on request.

## Supplementary Materials

The following supporting information can be downloaded at: https://www.mdpi.com/article/10.3390/ijms26030895/s1.

## Abbreviations

Abbreviation	Full Name
MALFD	Malonyl-CoA decarboxylase
MASH	Mast Cell Activation Syndrome
MCS	Methionine- and choline-supplemented diet
MCD	Methionine- and choline-deficient diet
THC	Tetrahydrocurcumin
OCA	Obeticholic acid
TG	Triglycerides
MDA	Malondialdehyde
ALT	Alanine aminotransferase
AST	Aspartate aminotransferase
FFA	Free fatty acid
PE	Phosphatidylethanolamine
12,13-EpOME	12,13-Epoxyoctadecadienoic Acid
9,10-EpOME	9,10-Epoxyoctadecadienoic Acid
9,10-DiHOME	9,10-Dihydroxyoctadecanoic Acid
14(S)-HDHA	14(S)-Hydroxydocosahexaenoic Acid
LPC	Lysophosphatidylcholine
LPE	Lysophosphatidylethanolamine
PC	Phosphatidylcholine
PI	Phosphatidylinositol
PA	Phosphatidic acid
CE	Cholesteryl ester
TxB3	Thromboxane B3
SM	Sphingomyelin
23-DCA	23-deoxycholic acid
β-MCA	Beta-muricholic acid
ω-MCA	Omega-muricholic acid
7-KDCA	7-ketodeoxycholic acid
UCA	Ursocholic acid
CA	Cholic acid
HDCA	Hyodeoxycholic acid
TDCA	Taurodeoxycholic acid
Cyp7a1	Cytochrome P450 7A1
Cyp27a1	Cytochrome P450 27A1
Mrp2	Multidrug Resistance-associated Protein 2
Bsep	Bile Salt Export Pump
Ntcp	Na⁺/taurocholate-cotransporting polypeptide
Oatp1b2	Organic Anion-Transporting Polypeptide 1B2
Srebp1c	Sterol Regulatory Element-binding Protein 1c
Fas	Fatty acid synthase
Scd1	Stearoyl-CoA Desaturase 1
Acc1	Acetyl-CoA Carboxylase 1
