# Supplementary material for: Tetrahydrocurcumin Alleviates Metabolic Dysfunction-Associated Steatohepatitis in Mice by Regulating Serum Lipids, Bile Acids, and Gut Microbiota
Source: Int J Mol Sci. 2025 Jan 22;26(3):895. doi: 10.3390/ijms26030895 (PMC11816436; doi:10.3390/ijms26030895)
Supplement: Supplementary file 1 [file ijms-26-00895-s001.zip › ijms-3395162-supplementary.pdf]

THC improved OA and PA-induced lipid accumulation and oxidative stress in hepatocytes

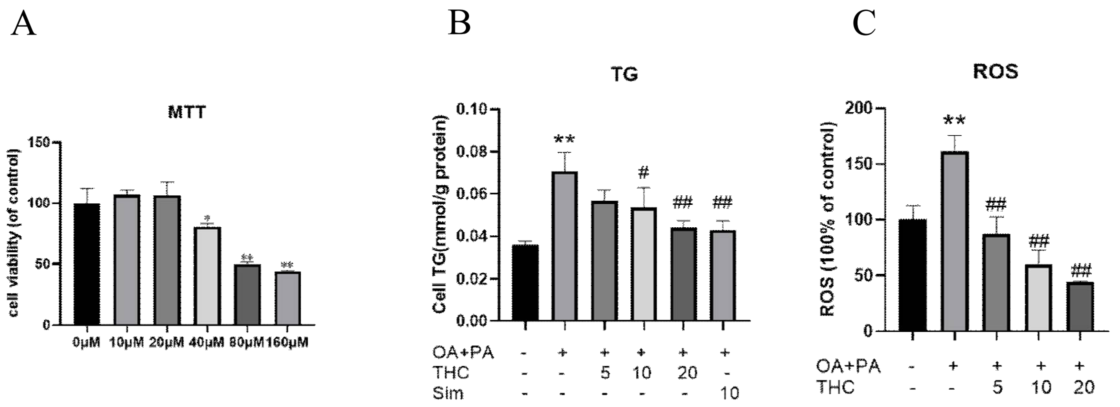

Figure S1 The effect of THC on NCTC-1469 cells induced by Oleic Acid（OA） and Palmitic Acid （OA）; (A) Effect of THC on NCTC-1469 cell survival; (B) Effect of THC on TG levels in OA- and PA-induced NCTC-1469 cells; (C) Effect of THC on ROS levels in OA- and PA-induced NCTC-1469 cells *p < 0.05, **p < 0.01 versus Control, $^{\#}p$ < 0.05, $^{\#\#}p$ < 0.01 versus Model group.
